# *Erigeron annuus* Protects PC12 Neuronal Cells from Oxidative Stress Induced by ROS-Mediated Apoptosis

**DOI:** 10.1155/2020/3945194

**Published:** 2020-01-06

**Authors:** Ji Yeon Lee, Jeong-Yong Park, Dong Hwi Kim, Hyung Don Kim, Yun-Jeong Ji, Kyung Hye Seo

**Affiliations:** ^1^Department of Herbal Crop Research, National Institute of Horticultural & Herbal Science, Eumsung 27709, Republic of Korea; ^2^Department of Food Science and Biotechnology, Chungbuk National University, Chungbuk 28644, Republic of Korea

## Abstract

Reactive oxygen species (ROS), associated with oxidative stress, are involved in many biological processes such as apoptosis, necrosis, and autophagy. Oxidative stress might induce neuronal damage via ROS generation, causing neurodegenerative diseases. *Erigeron annuus* (EA) has antioxidant properties and could protect neurons from oxidative stress. In this study, we investigated the protective effect of the aerial parts (EAA) and flowers (EAF) from EA on ROS-mediated apoptosis in pheochromocytoma 12 cells. We quantified 18 types of phenolic compounds using high-performance liquid chromatography. Pretreatment of the cells with EAA and EAF attenuated ROS generation and induced the expression of antioxidant enzymes such as superoxide dismutase 2, catalase, and glutathione peroxidase. In addition, EAF reduced the expression of apoptotic proteins such as Bax/Bcl-xL, caspase-3, and caspase-8 to a greater extent than that with EAA. These results suggested that the protective effect of EAF against oxidative stress-induced apoptosis might be due to the prevention of ROS generation mediated by oxidative enzymes.

## 1. Introduction

Reactive oxygen species (ROS) play an important role in regulating normal physiological and developmental functions such as cell cycle progression, proliferation, differentiation, migration, and cell death. ROS are generated in the mitochondria as byproducts of cellular metabolism [1]. Oxidative stress induced by ROS, such as superoxide (O_2_) or hydrogen peroxide (H_2_O_2_), has been associated with several pathologies and diseases such as diabetes, arthrosis, and Alzheimer's and Parkinson's diseases [2]. When the production of ROS surpasses the cellular antioxidant capacity, damage to macromolecules such as protein and DNA contributes to cell toxicity or apoptosis directly or indirectly [1–3]. Among the enzymes that are involved in ROS generation, catalase (CAT) and glutathione peroxidase (GPx) convert H_2_O_2_ to H_2_O; meanwhile, superoxide dismutase (SOD) converts O_2_ to H_2_O_2_ [4, 5]. Additionally, the SOS response has the effect of eliminating the ROS reaction, only if its enzymatic activity interacts with that of CAT and/or CAT.

Apoptosis is controlled by extrinsic and intrinsic pathways (mitochondrial pathway) [6]. ROS-mediated mechanisms drive apoptosis through intrinsic pathways to regulate cell death [3]. The intrinsic apoptosis pathway consists of intracellular signaling between proapoptotic proteins. For example, the Bcl-2 family includes proteins such as the antiapoptotic activator Bcl-xL and the proapoptotic effector Bax, which interacts with other proteins [7]. Additionally, overexpression of the antiapoptotic protein Bcl-xL, which appears to be bound to the mitochondrial membrane, can block apoptosis [8]. Conversely, Bax causes apoptosis by inducing the release of cytochrome-*c*, a key component of the mitochondria electron transport chain, into the cytoplasm [8, 9]. This intrinsic pathway induces caspase-independent cell death, as it is controlled by Bcl-2 family proteins such as Bax and Bcl-xL [7]. In contrast, procaspase-8 is the apical caspase in the extrinsic pathway, for which activation occurs within the DISC (death-inducing signaling complex), and proceeds by directly activating procaspase-3 [6]. Procaspase-3 exists in the cytosol as an inactivated dimer, and the activation of procaspase-3 takes places after the cleavage of caspase-8 [10]. Cleaved caspase-3 acts as a direct executioner of apoptosis [10].

*Erigeron annuus* (EA), which belongs to the Asteraceae family, has white flowers and is commonly found in grasslands and roadsides. In addition, EA has traditionally been used as a medicinal plant for dyspepsia, abdominal pain, urine bleeding, and hypoglycemic effects [11]. Many compounds such as flavanone, erigeroflavanone, sesquiterpenoids, ergosterol peroxide, caffeic acid, and pyromeconic acid can be derived from the aerial part (EAA) and flowers (EAF) of EA [12–17]. It has been reported that these compounds have several activities such as reductase inhibitory in aldose, antiatherosclerotic, neuroprotective, antioxidant, and cytoprotective effects [12, 14–17]. Although several studies have demonstrated the effect of EA as an antioxidant and neuroprotective agent, studies on its effect against damage to neuronal cells due to oxidative stress are scarce. In this study, we demonstrated that EAA and EAF can effectively block the intrinsic and extrinsic apoptosis pathways via ROS-mediated signaling. Our data suggest that EAA and EAF could inhibit ROS mediated-apoptosis in PC12 cells under oxidative stress by upregulating the expression of antioxidant enzymes and downregulating apoptotic proteins.

## 2. Materials and Methods

### 2.1. Chemicals, Antibodies, and Apparatus

All reagents were purchased from Sigma Aldrich (Saint Louis, MO, USA), unless otherwise indicated. CellTiter 96® AQ_ueous_ One Solution (MTS) was obtained from Promega (Madison, WI, USA). Pheochromocytoma (PC12) cells were purchased from the ATCC (Manassas, VA, USA). All cell culture reagents were obtained from Gibco (Burlington, ON, Canada). Radio immunoprecipitation (RIPA) cell lysis buffer was purchased from GenDepot (Katy, TX, USA). Bradford and enhanced chemiluminescence (ECL) reagents for protein assays were from Bio-Rad (CA, USA). All antibodies were from Abcam (Cambridge, UK), unless otherwise stated. Antibodies against *β*-actin, cleaved caspase-8, and caspase-8 were purchased from Santa Cruz (Dallas, TX, USA). Meanwhile, anticleaved caspase-3 was purchased from Cell Signaling (Danvers, MA, USA). Evaporation was conducted using an evaporator system under reflux *in vacuo* from BÜCHI (Sankt Gallen, Swiss). Multimode-plate reading was performed with a Synergy H1 Hybrid Reader from BioTek Instruments (Winooski, VT, USA). The confocal microscope for fluorescent imaging was purchased from Zeiss (Oberkochen, German). Protein expression levels were assessed with a chemiluminator from Davinch-K (Seoul, Korea). Analysis was performed using a high-performance liquid chromatography (HPLC) 2790/5 system equipped with a photodiode array (PDA) 2996 from Waters (Milford, MA, USA). The INNO column was obtained from Young Jin Biochrom Co., Ltd., (Seoul, Korea). Acetonitrile and water labeled as HPLC grade solvents were purchased from Fisher Scientific Ltd., (Sunnyvale, CA, USA).

### 2.2. Plant Materials, Extraction, and Yields

EA samples were collected from Eumsung (Chungcheongbuk-do, Korea) in 2018. The plant material was identified by Jeong Hoon Lee, PhD. (Department of Herbal Crop Research), and the voucher specimen (NIBRVP0000456433) was deposited at National Institute of Biological Resources. They were then divided into two groups, the aerial part (stem and leaves, EAA) and flower part (EAF). For the preparation of extracts, EAA and EAF (100 g, each) were grinded, sifted through a testing sieve (aperture 1.40 mm, wire 0.71 mm), and extracted three times with 70% ethanol at a 1 : 10 (*v* : *v*) ratio for 24 h, at room temperature. After filtration, all extracts were evaporated *in vacuo*, freeze-dried (20 mTorr, −40°C, 1 week), and stored at −80°C. Extraction yields were calculated as previously described [18] as follows: yields (%) = total extract dried weight/raw material weight × 100.

### 2.3. Analysis of Antioxidant Components

#### 2.3.1. Determination of Total Phenolic Contents (TPC)

TPCs of extracts were determined by the Folin–Denis's phenol method, with slight modifications [19]. Specifically, 500 *μ*L of each extract (2 mg/mL) was mixed with 50 *μ*L of 1 N Folin–Ciocalteu reagent for 3 min. Then, 100 *μ*L of 20% sodium carbonate solution was added. After 1 h, the absorbance was measured at 725 nm using a multimode-plate reader. TPC was calculated from the calibration curve using gallic acid, and the results were expressed as gallic acid equivalents (*y* = 0.064*x* + 0.024).

#### 2.3.2. Determination of Total Flavonoid Contents (TFC)

TFCs of each extract were determined by the aluminum chloride colorimetric method, with slight modifications [20]. Briefly, 150 *μ*L of each extract (2 mg/mL) was mixed with 10 *μ*L of 10% aluminum chloride solution, 10 *μ*L of 1 M potassium acetate, and 280 *μ*L of distilled water. The mixture was kept at room temperature for 30 min and then measured at 415 nm using a multimode-plate reader. TFC was expressed as quercetin equivalents (*y* = 0.001*x* + 0.015), which reflected the amount of quercetin (*μ*g/mL).

### 2.4. Antioxidant Activity Assay

#### 2.4.1. ABTS^+^ Radical Scavenging Assay

ABTS^+^ (2,2′-azino-bis 3-ethylbenzothiazolin-6-sulfonic acid) radical scavenging activity was measured as described by Van den Berg, with some modifications [21]. ABTS^+^ solutions containing 7.4 mM 2,2′-azino-bis (3-ethylbenzothiazoline-6-sulfonic acid) ammonium salt and 2.6 mM potassium persulfate were prepared in distilled water for 24 h. The absorbance of the solution was adjusted to 0.70 ± 0.05 at 732 nm. Next, 20 *μ*L samples were mixed with 180 *μ*L of ABTS^+^ solution and incubated for 30 min in the dark at room temperature. Absorbance values of ABTS radicals were measured for the different samples using a multimode-plate reader at 732 nm. The following samples were tested: Treatment, Blank1, Blank2, and Control corresponded to the sample, H_2_O, H_2_O + sample, respectively. An ABTS radical scavenging assay (RSA) was performed for each concentration according to the following equation: ABTS^+^ RSA (%) = [(*A*_control_ − *A*_Blankl1_) − (*A*_Treatment_ − *A*_Blank2_)/*A*_control_ − *A*_Blankl1_] × 100. Additionally, the half-maximal inhibitory concentration (IC_50_) was calculated for each extract.

#### 2.4.2. DPPH Radical Scavenging Assay

DPPH (2,2-diphenyl-1-picryllydrazyl) radical scavenging activity was measured according to the Bondet method, with some modifications [22]. DPPH solution containing 300 *μ*M of DPPH in 95% ethanol was prepared. The absorbance of the solution was adjusted to 1.00 ± 0.05 at 515 nm. Next, 25 *μ*L of samples was mixed with 225 *μ*L of DPPH solution and incubated for 30 min in the dark covered with aluminum foil at room temperature. Absorbance was measured using a multimode-plate reader at 515 nm. Among the samples tested were the following: Treatment, Blank1, Blank2, and Control corresponding to the sample, H_2_O, and H_2_O + sample, respectively. DPPH radical scavenging activity (RSA) was calculated for each concentration using the following equation: DPPH RSA (%) = [(*A*_control_ − *A*_Blankl1_) − (*A*_Treatment_ − *A*_Blank2_)/*A*_control_ − *A*_Blankl1_] × 100. The half-maximal inhibitory concentration (IC_50_) was also calculated for each extract.

### 2.5. HPLC Analysis of Antioxidant Compounds

#### 2.5.1. Sample Preparation for HPLC

To investigate phenolic compounds in EAA and EAF (5 g), the modified method of Ahn et al. was applied [23]. Each extract was redissolved in water and fractionated with ethyl acetate/ether (1 : 1 = *v*/*v*) to obtain the phenol-rich fraction. Each fraction was concentrated under reduced pressure, dissolved in methanol (10 mg/mL each), filtered through a 0.22 *μ*M polyvinylidine difluoride (PVDF) membrane, and analyzed by HPLC.

#### 2.5.2. HPLC for Phenolic Compound Analysis

Antioxidant components and phenolic compounds were analyzed by a Waters HPLC 2790/5 system equipped with PDA by Waters 2996. HPLC separation of compounds for qualitative and quantitative analysis was performed using a reverse phase system with an INNO column at 35°C. Homogentisic acid, gallic acid, protocatechuic acid, chlorogenic acid, (+)-catechin, caffeic acid, phloretic acid, p-coumaric acid, ferulic acid, veratric acid, salicylic acid, naringin, hesperidin, quercetin, cinnamic acid, naringenin, kaempferol, and hesperidin were used as phenolic compound standards. All standards were prepared in methanol and filtered through a 0.22 *μ*M PVDF membrane. The mobile phase consisted of solvent A (0.5% acetic acid in water) and solvent B (0.5% acetic acid in acetonitrile). The gradient program with the mobile phase was modified [23]. The elution program was as follows (B %): 8–10%, 2 min; 10–30%, 27 min; 30–90%, 50 min; 90–100%, 51 min; 100%, 60 min; 100–8%, 62 min; and 8%, 70 min. The injection volume was 10 *μ*L, the flow rate was 1.0 mL/min, and phenols were detected at 280 nm using UV light.

### 2.6. Cell Culture

PC12 cells were cultured in Dulbecco's modified Eagle's medium (DMEM), supplemented with 10% fetal bovine serum (FBS) and 1% penicillin-streptomycin at 37°C in a 5% CO_2_ incubator. The medium was replaced every 2 days for subculture. Cells from passages 5–10 were used in all experiments.

### 2.7. Intracellular ROS Generation

Intracellular ROS generation was measured by a modified dichloro-dihydro-fluorescein diacetate (DCFH-DA) method [24]. PC12 cells were cultured with EAA and EAF in black 96-well plates at a density of 1.0 × 10^4^ cells/well. After 24 h, the culture cells were treated with 50 *μ*M H_2_O_2_ in SFM for 20 min and then with 20 *μ*M DCF-DA in serum-free medium for 30 min; samples were then washed, and 100 *μ*L Dulbecco's phosphate buffered saline (DPBS) was added to each well. Vitamin C (10 *μ*g/mL) was used as a positive control. Vitamin C is well known as an antioxidant and is widely used as a positive control for antioxidant studies. The fluorescence was measured with a multiplate reader at 485 nm/535 nm (excitation/emission). In addition, fluorescent micrographs were obtained with a confocal microscope.

### 2.8. Preparation of Protein Samples

PC12 cells were pretreated with EAA and EAF (50, 100, and 200 *μ*g/mL) for 24 h and with H_2_O_2_ (50 *μ*M) for 20 min. Then, cells were rinsed, scraped off, and collected with DPBS on ice. After centrifugation at 3000 rpm, the DPBS was removed completely, and cells were lysed with RIPA buffer for total protein extraction, following the manufacturer's instructions. Protein amounts were determined using the Bradford assay. Protein samples were mixed with 5x loading buffer, boiled for 5–10 min, and stored at −80°C.

### 2.9. Western Blotting

Western blotting was performed with 10–15% tris-HCl gels for sodium dodecyl sulfate-polyacrylamide gel electrophoresis (SDS-PAGE). The proteins were transferred onto PVDF membranes, washed three times, and subsequently incubated with primary antibodies (at a 1 : 1000 dilution) at 4°C overnight. The membranes were washed three times again and incubated with horseradish peroxidase-conjugated secondary antibody (at a 1 : 2000 dilution) for 1 h at room temperature [25]. Protein expression was visualized using the ECL reagent, following the manufacturer's instructions.

### 2.10. Statistical Analysis

All experimental data were expressed as means ± standard deviations (SDs) of three independent experiments. The statistical analyses in this study were performed using a one-way analysis of variance (ANOVA) with a Duncan's test, *t*-tests and correlation analysis based on Pearson's correlation coefficient (Statistical Package for the Social Sciences, ver. 21.0 for Window ver. 10).

## 3. Results

### 3.1. Antioxidant Components and Activities of EAA and EAF

It has been reported that 70% ethanol is the most effective solution to extract triterpenoids from *Jatropha curcas* leaves and phenols from *Moringa oleifera* and *Curcuma longa* extractions [26]. Therefore, the extraction of EAA and EAF rendered yields of 25.16% and 25.84%, respectively (Table 1). The compound contents were analyzed by measuring TPC and TFC. The concentrations of the phenolic compounds, namely, TPC and TFC, with antioxidant activity [27] were determined for each EA part, specifically for EAA (9.8 and 20.8 mg/g) and EAF (8.9 and 10.1 mg/g) (Table 1). Moreover, we calculated the levels of DPPH and ABTS^+^, indicative of antioxidant activity, by simple colorimetric methods [28]. The antioxidant activities of EAA and EAF were examined using ascorbic acid (AA) as the positive control [29]. IC_50_ values of EAA and EAF were 11.0 and 17.5 *μ*g/mL for ABTS^+^ and 114.0 and 112.2 5 *μ*g/mL for the DPPH-scavenging effect, respectively (Table 1).

### 3.2. Quantification of Phenolic Compounds from EAA and EAF by HPLC

**Figure 1:**
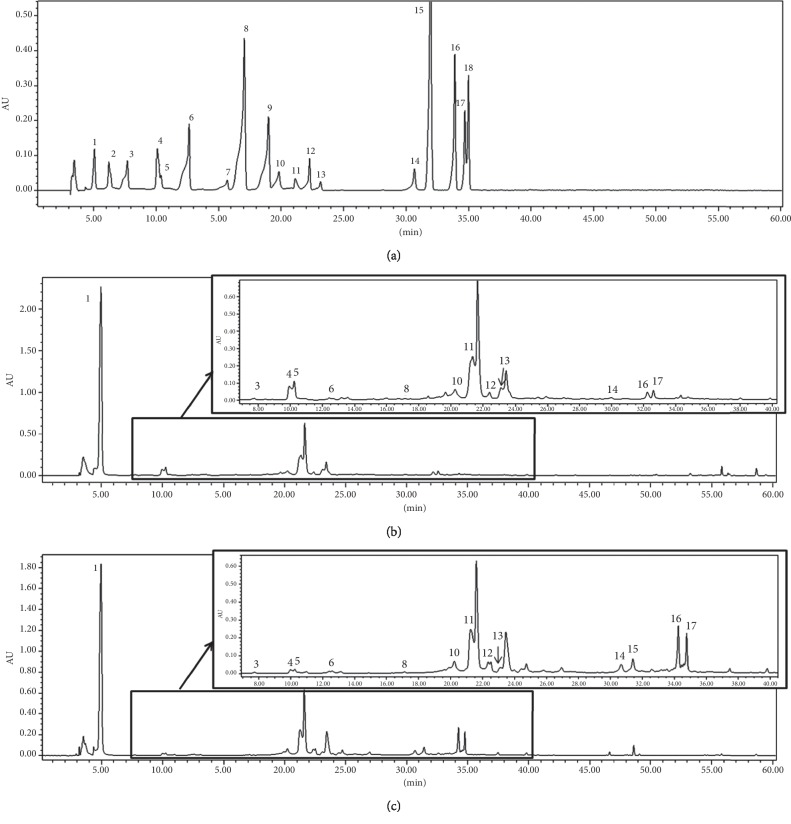
The chromatograms of phenolic compounds from EAA and EAF based on HPLC analysis. Peak identification: 1: homogentisic acid, 2: gallic acid, 3: protocatechuic acid, 4: chlorogenic acid, 5: (+)-catechin, 6: caffeic acid, 7: phloretic acid, 8: *p*-coumaric acid, 9: ferulic acid, 10: veratric acid, 11: salicylic acid, 12: naringin, 13: hesperidin, 14: quercetin, 15: cinnamic acid, 16: naringenin, 17: kaempferol, and 18: hesperidin. Chromatogram of (a) standard solution used for phenolic compound analysis (100 *μ*g/mL each), (b) EAA (10 mg/mL), and (c) EAF (10 mg/mL). EAA: *Erigeron annuus* aerial parts; EAF: *E. annuus* flowers.

Phenolic compounds, which are known to be responsible for the beneficial effects of EAA and EAF, were analyzed by HPLC using a PDA detector. For the determination of phenolic compounds, EAA and EAF were reextracted from phenol-rich fractions. The chromatograms of phenolic compounds and phenol-rich fractions of EAA and EAF are shown in Figure 1. The homogentisic acid peak was the highest for both EAA and EAF and the peak of kaempferol was higher with EAF than with EAA. In addition, gallic, phloretic, and ferulic acids were not detected, but veratric and cinnamic acids were detected specifically in EAF. Phenolic compound contents in EAA and EAF were identified based on the calibration curve (Tables 2 and 3). The total phenolic compound (TP) contents of EAF (97.32 mg/g, 1.43-fold), as analyzed by HPLC, were higher than those of EAA, and these results were similar for TPC and TFC. The contents of homogentisic acid (1.11-fold), protocatechuic acid (1.18-fold), (+)-catechin (3.03-fold), naringin (2.36-fold), and naringenin (1.79-folds) were higher in EAA than in EAF. Conversely, the contents of chlorogenic acid (2.06-fold), *p*-coumaric acid (1.47-fold), salicylic acid (5.89-fold), hesperidin (2.68-fold), quercetin (3.60-fold), and kaempferol (21.31-fold) were higher in EAF than in EAA. As a result, EAA and EAF might have higher scavenging effects because of these phenolic compounds.

### 3.3. EAA and EAF Reduce Intracellular ROS Generation in PC12 Cells

**Figure 2:**
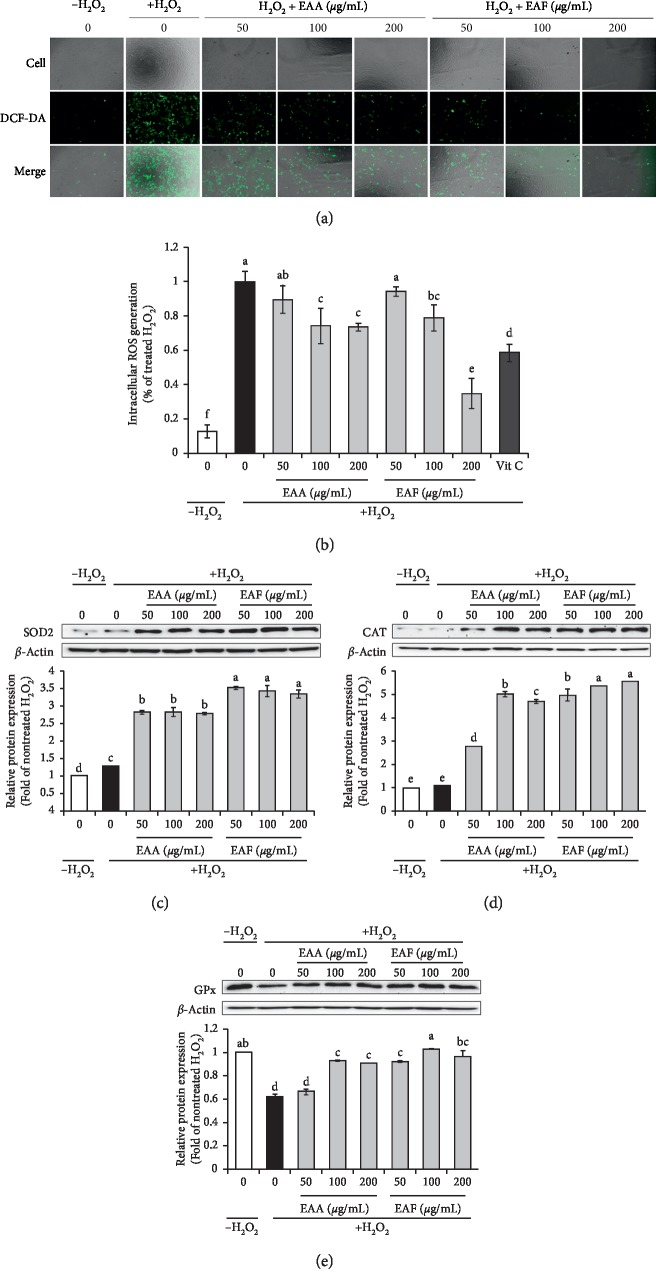
EAA and EAF protect PC12 cells from H_2_O_2_-induced oxidative injury. (a) The cells were treated with EAA and EAF (50, 100, and 200 *μ*g/mL) for 24 h and then treated with DCFH-DA (20 *μ*M) for 30 min. The intracellular levels of ROS were visualized using fluorescent confocal microscopy. (b) Quantitative measurement of intracellular ROS generation. (c–e) Cells were treated with EAA and EA (50, 100, and 200 *μ*g/mL) for 24 h. Vitamin C (ascorbic acid) was positive control (50 *μ*g/mL). The expression levels of SOD2 (c), CAT (d), and GPx (e) were detected by western blot analysis in PC12 cells. All protein expression levels were quantified by normalizing to *β*-actin levels. All columns are means ± SD (*n* = 3). Means with different letters on the all-color columns were significantly different at *p* < 0.05 based on Duncan's test. EAA: *Erigeron annuus* aerial parts; EAF: *E. annuus* flowers.

ROS production was analyzed by staining with DCFH-DA and detecting and quantitatively measuring the fluorescence by confocal microscopy (Figure 2(a)). Incubation of PC12 cells with H_2_O_2_ led to an increase in DCF fluorescence intensity, which was proportional to the amount of ROS generated. However, after treatment with EAA and EAF, the green fluorescence signal of H_2_O_2_-treated cells decreased compared with that observed for the controls. The addition of vitamin C (50 *μ*g/mL) served as a positive control with an inhibition rate of approximately 60%. EAF (200 *μ*g/mL) resulted in a more pronounced reduction than the positive control. These results showed that ROS generation was significantly elevated in a dose-dependent manner (Figure 2(b)). Intracellular ROS generation was reduced to a greater extent after pretreating PC12 cells, specifically up to 7.87-fold. The levels of intracellular ROS generated in cells treated with EAA and EAF (50, 100, and 200 *μ*g/mL) decreased in a concentration-dependent manner compared with those in cells treated with H_2_O_2_ only (EAA, 74.27 and 73.62%; EAF, 78.81 and 34.92%).

### 3.4. EAA and EAF Reduce Levels of Oxidative Stress-Related Proteins

The antioxidant protein levels of SOD2, CAT, and GPx correlate with reductions in ROS [30]. Therefore, we investigated the effect of EAA and EAF on the expression levels of these oxidative proteins in H_2_O_2_-treated PC12 cells (Figures 2(c)–2(e)). SOD2 activity was significantly increased by H_2_O_2_ (1.28-fold) and further increased in the presence of EAA and EAF (2.78–2.83- and 3.35–3.52-fold, respectively) in H_2_O_2_-treated PC12 cells. In contrast, H_2_O_2_ significantly maintained or reduced the levels of the antioxidant enzymes CAT and GPx (1.07- and 0.61-fold, respectively). Meanwhile, EAA and EAF enhanced the activities of CAT (2.79–4.70- and 4.96–5.54-fold, respectively) and GPx (0.66–0.92- and 0.92–1.02-fold, respectively) in H_2_O_2_-treated PC12 cells.

### 3.5. Inhibitory Effects of EAA and EAF on Apoptosis in H_2_O_2_-Treated PC12 Cells

**Figure 3:**
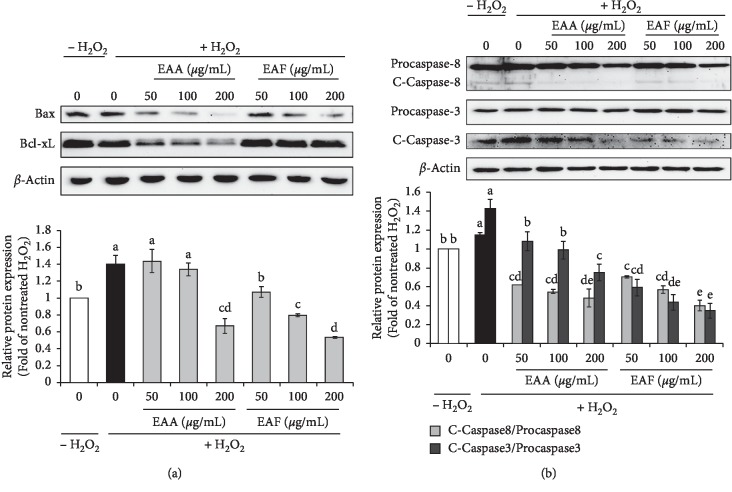
EAA and EAF protect PC12 cells from H_2_O_2_-induced oxidative apoptosis. (a) Protein expression related to the intrinsic pathway of apoptosis (Bax/Bcl-xL). (b) Protein expression related to the extrinsic pathway (cleaved- (C-) pro-caspase-8 and -3). Cells were treated with EAA and EA (50, 100, and 200 *μ*g/mL) for 24 h. Protein expression levels were detected by western blot analysis in PC12 cells. Protein expression was quantified based on normalized *β*-actin levels. All columns are means ± SD (*n* = 3). Means with different letters on the all-color column were significantly different at *p* < 0.05 based on Duncan's test. EAA: *Erigeron annuus* aerial parts; EAF: *E. annuus* flowers.

We finally investigated whether ROS affects EAA- and EAF-induced apoptosis based on the two major pathways (intrinsic and extrinsic). To determine the effect of EAA and EAF on apoptosis in H_2_O_2_-treated PC12 cells, protein levels of components of the intrinsic and extrinsic pathways were evaluated by western blotting. Results from the intrinsic pathway are shown in Figure 3(a). Specifically, treatment with H_2_O_2_ only upregulated the expression of Bax/Bcl-xL compared with that in untreated cells. Exposure of H_2_O_2_-treated PC12 cells to EAA and EAF reduced the expression of Bax/Bcl-xL (0.09–0.67-fold) in a dose-dependent manner. Results of the extrinsic pathway are shown in Figure 3(b). In this investigation, the cells treated with H_2_O_2_ only showed increased levels of cleaved-caspase8 and cleaved-caspase 3 (1.20- and 1.31-fold, respectively) compared with those in untreated cells. However, our results showed that EAA and EAF reduced pro- and cleaved-caspase8 and cleaved-caspase 3 levels in a dose-dependent manner.

## 4. Discussion

Air pollution caused by ambient air particulate matter such as diesel exhausts particles, and nitrate induces oxidative stress that can trigger ROS [31, 32]. ROS generates toxic byproducts that cause damage to the cell by producing oxidative stress; however, the cell expresses several antioxidant enzymes to protect against this effect [1]. The overexpression of ROS causes an imbalance between oxidant production and antioxidant capacity and leads to the onset of neurodegenerative diseases such as Alzheimer's and Parkinson's diseases [2]. ROS can decrease through nonenzymatic and enzymatic reactions [30]. Nonenzymatic antioxidants such as phenolic acids, flavonoids, and other compounds increase the activity of glutathione peroxidase GSH (which reduces H_2_O_2_ to H_2_O) [30].

Phenolic compounds are present in various medicinal plants [33]. The phenolic hydroxyl groups in phenolic compounds play an important role in antioxidant properties by donating hydrogens and scavenging radicals [34]. Therefore, we identified antioxidants in the phenol-rich fractions of EAA and EAF by HPLC analysis. Linearity of the calibration curve with a correlation coefficient >0.99 was obtained from the plot of a minimum of five standard concentrations (10, 25, 50, 100, and 200 *μ*g/mL) by using the least square method (Table 3) [35]. Homogentisic and salicylic acids, which were abundant in both EAA and EAF, confer beneficial effects such as the inhibition of oxidation, anti-inflammation [36–38], and disease tolerance in plants [39]. Kaempferol, which was significantly present in EAF, exerts other beneficial effects such as anticancer cell proliferation, *in vitro* antioxidation, and the inhibition of autophagy [40–43]. These phenolic and flavonoid compounds produced by photosynthesis are stored in plant leaves and accumulate in the vacuoles of flowers [44]. Indeed, phenols and flavonoids in plants have antioxidant effects by transferring hydroxyl groups to the radicals in ABTS^+^ and DPPH and participating in electron delocalization, thus providing stability [45]. Our results suggested that the antioxidative effects of EAA and EAF might be due to their TPC and TFC contents. Importantly, antioxidant contents and activities were not significantly different between EA parts. However, EAA and EAF have stronger antioxidant activities than AA in ABTS^+^ scavenging (3.1- and 1.9-fold, respectively). The correlation between antioxidant activities (ABTS^+^ and DPPH) and the content of TPs and phenolic compounds (homogentisic acid, salicylic acid, and kaempferol) in EAA and EAF was also investigated (Table 4). The antioxidant effect of ABTS^+^ was significantly correlated with TPAs (−0.921), salicylic acid (−0.975), and kaempferol (−0.0695). These results showed that the antioxidant effects on ABTS^+^ and DPPH were due to TPs, salicylic acid, and kaempferol contained in EAA and EAF.

Enzymatic antioxidants, which decrease ROS generation (such as SOD2 (which reduces O_2_^−^ to H_2_O_2_) and CAT and GPx (which reduce H_2_O_2_ to H_2_O)), are usually secreted into the cytosol to protect tissue damage due to oxidative stress [3, 30]. In addition, H_2_O_2_ generates intracellular ROS which regulate apoptosis [3]. To measure the inhibition of intracellular ROS generation in the presence of H_2_O_2_, we tested the application of various concentrations of EAA and EAF (50, 100, and 200 *μ*g/mL) to PC12 cells after treating the cells with 50 *μ*M of H_2_O_2_ for 20 min, as those conditions resulted in the highest ROS generation (data not shown). Our results showed that intracellular ROS generation was reduced in a dose-dependent manner after pretreatment with EAA and EAF, compared with that in PC12 cells only treated with H_2_O_2_ (Figures 2(a) and 2(b)). The maintenance of homeostasis in multicellular organisms depends on complex networks of intracellular signals [1]. Cell organelles generate intracellular ROS via oxidative stress injury [2]. When ROS are overproduced in cells, they cause various diseases in humans, including neurological diseases [1, 2]. However, ROS can be inhibited by antioxidant components [2]. For example, caffeic acid isolated from *Erigeron annuus* leaves reduced H_2_O_2_-induced ROS in PC12 cells. In addition, salicylic acid increases levels of antioxidant enzymes that reduce ROS generation such as CAT, SOD, and GPx. Meanwhile, kaempferol was found to protect HIT-T15 cells from oxidative damage [15, 42, 46]. Our results showed higher levels of TPC, TPF, and phenolic compounds, which have antioxidant effects, following the treatment of PC12 cells with EAF, compared with those observed with EAA treatment. Moreover, among TPs, salicylic acid and kaempferol were significantly correlated with ROS generation as shown in Table 4 (−0.965 and −0.976). Our study indicated that EAF contained more antioxidant compounds than EAA, and thus EAF further prevented oxidation in H_2_O_2_-treated PC12 cells.

Apoptosis-induced ROS comprises two major pathways, the intrinsic and extrinsic [6]. The proapoptotic members (Bcl-xL and Bax) release cytochrome *c* to the mitochondrial membrane through the intrinsic pathway [3]. In the extrinsic pathway, cleaved-caspase-8 cleaves and activates procaspase-3 [10]. Through these two pathways, caspase 3 directs cell death directly [10]. In our investigation, EAA and EAF reduced the levels of apoptosis-activating factors such as caspase-3 and caspase-8, as well as the Bax/Bcl-xL ratio. Specifically, these apoptotic factors were further decreased with EAF treatment compared with that with EAA. Antioxidant enzymes are known to prevent apoptosis via ROS and directly reduce ROS generation [3, 30]. EAF reduced apoptotic protein levels, and its antioxidant effect was higher than that of EAA, even in the presence of nonenzymatic antioxidants such as phenolic acid and flavonoid. Further, EAF downregulated the intracellular apoptosis pathways induced by ROS generation (Figures 2(c)–2(e) and 3).

## 5. Conclusions

The present study demonstrates that EAA and EAF have neuroprotective effects on PC12 cells. EAA and EAF exerted protective effects against apoptosis by targeting oxidant and antioxidant agents such as SOD2, CAT, and the GPx system. Specifically, EAF contained abundant phenolic compounds and had a higher antioxidant effect than EAA. EAF could exert its antiapoptotic effect by reducing the number of ROS and enzymatic factors. Therefore, it could be a good source of natural antioxidants to prevent neurodegenerative diseases arising from oxidative stress-induced neuronal damage by regulating the expression of apoptotic proteins.

## Figures and Tables

**Table 1:** Antioxidant components, activities, and yields of EAA and EAF.

Samples^a^	TPC^b^ (GAE mg/g)	TFC^c^ (QUE mg/g)	ABTS^+^ (IC_50_, *μ*g/mL)	DPPH (IC_50_, *μ*g/mL)	Yields (%)
EAA	9.8 ± 0.1b	8.9 ± 0.6b	11.0 ± 0.6b	114.0 ± 5.4a	25.16 ± 0.86a
EAF	20.8 ± 0.1a	10.1 ± 0.1a	17.5 ± 0.6b	112.2 ± 2.9a	25.84 ± 2.29a
AA	—	—	34.1 ± 0.3a	10.2 ± 0.3b	—

^a^EAA: aerial parts of *Erigeron annuus*; EAF: flowers of *Erigeron annuus*; AA: ascorbic acid. ^b^TPC: total phenol contents. ^c^TFC: total flavonoid contents. All values are means ± SD. Means with different letters on the same column are significantly different at *p* < 0.05 by *t*-tests (for TPC, TFC, and yields) and Tukey's test (for ABTS^+^ and DPPH).

**Table 2:** Phenolic compounds contents of EAA and EAF analyzed by HPLC (*n* = 3).

Number	Phenolic compounds	Contents (mg/g dried weight)	
		EAA^a^	EAF^b^
1	Homogentisic acid	53.75 ± 1.51a	48.42 ± 4.15a
2	Gallic acid	N.D.^c^	N.D.
3	Protocatechnic acid	0.73 ± 0.01e	0.62 ± 0.00d
4	Chlorogenic acid	2.02 ± 0.02c	0.98 ± 0.01d
5	(+)-Catechin	2.09 ± 0.26c	0.69 ± 0.02d
6	Caffeic acid	0.64 ± 0.01e	0.92 ± 0.02d
7	Phloretic acid	N.D.	N.D.
8	*p*-Coumaric acid	0.61 ± 0.02e	0.90 ± 0.02d
9	Ferulic acid	N.D.	N.D.
10	Veratric acid	N.D.	0.85 ± 0.03d
11	Salicylic acid	3.63 ± 0.18b	21.40 ± 1.48b
12	Naringin	1.74 ± 0.07cd	0.74 ± 0.03d
13	Hesperidin	1.00 ± 0.03de	2.69 ± 0.21d
14	Quercetin	0.52 ± 0.00e	1.88 ± 0.02d
15	Cinnamic acid	N.D.	0.89 ± 0.07d
16	Naringenin	0.54 ± 0.00e	0.42 ± 0.00d
17	Kaempferol	0.75 ± 0.01e	15.94 ± 1.47c
18	Hesperitin	N.D.	N.D.

Total contents		68.02 ± 1.90	97.21 ± 7.52

^a^EAA: aerial parts of *Erigeron annuus*, ^b^EAF: flowers of *Erigeron annuus*, ^c^N.D.: not detected. All values are means ± SD. Means with different letters on the same line (to analyze EAA and EAF) are significantly different at *p* < 0.05 by Duncan's test.

**Table 3:** Retention time and calibration curves of standards (*n* = 3).

Number	Phenolic compounds	Rt^a^ (min)	R^2b^	Calibration curve^c^ (*Y* = aX + *b*)
1	Homogentisic acid	5.07	1.00	*Y* = 15266*X* − 261028
2	Gallic acid	6.37	0.99	*Y* = 12344*X* − 257389
3	Protocatechnic acid	7.67	1.00	*Y* = 17658*X* − 339681
4	Chlorogenic acid	10.16	1.00	*Y* = 21148*X* − 461992
5	(+)-Catechin	10.26	1.00	*Y* = 3427.1*X* − 41775
6	Caffeic acid	12.49	1.00	*Y* = 45912*X* − 904096
7	Phloretic acid	15.58	0.99	*Y* = 6986.6*X* − 173117
8	*p*-Coumaric acid	16.95	1.00	*Y* = 109086*X* − 2095025
9	Ferulic acid	18.92	1.00	*Y* = 52036*X* − 996550
10	Veratric acid	19.76	1.00	*Y* = 12550*X* − 234385
11	Salicylic acid	21.13	1.00	*Y* = 5350*X* − 102288
12	Naringin	22.25	0.99	*Y* = 11740*X* − 158668
13	Hesperidin	23.12	1.00	*Y* = 3197*X* − 57859
14	Quercetin	30.61	1.00	*Y* = 11756*X* − 257156
15	Cinnamic acid	31.90	1.00	*Y* = 112897*S* − 2*E* + 06
16	Naringenin	33.88	0.99	*Y* = 47325*X* − 691049
17	Kaempferol	34.69	1.00	*Y* = 27333*X* − 605965
18	Hesperitin	34.97	0.99	*Y* = 31446*X* − 426773

^a^Rt: retention time; ^b^correlation coefficients for three data points in the calibration curve; ^c^where the *Y* and *X* are the peak area and concentration of the analyses (*μ*g/mL), respectively.

**Table 4:** Correlation analysis of antioxidant components and activities.

Factors^a^	ABTS	DPPH	TP	Homogentisic acid	Salicylic acid	Kaempferol	ROS
ABTS	1.000	−0.127	−0.921^*∗∗*^	−0.764	−0.975^*∗∗*^	−0.0968^*∗∗*^	0.929^*∗∗*^
DPPH		1.000	0.326	−0.056	0.255	0.300	−0.469
TP			1.000	−0.489	0.979^*∗∗*^	−0.982^*∗∗*^	−0.949^*∗∗*^
Homogentic acid				1.000	−0.656	−0.641	0.637
Salicylic acid					1.000	−0.999^*∗∗*^	−0.965^*∗∗*^
Kaempferol						1.000	−0.976^*∗∗*^
ROS							1.000

^a^Factors: ABTS and DPPH was analyzed by IC_50_ value; TP, total contents of phenolic compounds; ROS, intracellular reactive oxygen species. Significance was determined using SPSS by Pierson's correlation coefficient; ^*∗*^*p* < 0.05 and ^*∗∗*^*p* < 0.01.

## Data Availability

The data used to support the findings of this study are available from the corresponding author upon request.
